# Plasma Concentrations of Oral Ondansetron in Hospitalized Dogs Exhibiting Clinical Signs of Nausea

**DOI:** 10.3390/vetsci11030112

**Published:** 2024-03-03

**Authors:** Kristin M. Zersen, Angela Molli, Brooke G. Weisbeck, Samantha Fedotova, Jessica M. Quimby, Daniel L. Gustafson, Sarah B. Shropshire

**Affiliations:** 1 Department of Clinical Sciences, College of Veterinary Medicine and Biomedical Sciences, Colorado State University, Fort Collins, CO 80523, USA; kristin.zersen@colostate.edu (K.M.Z.); angela.molli22@alumni.colostate.edu (A.M.); brooke.gallagher@colostate.edu (B.G.W.); samanthafedotova@gmail.com (S.F.); daniel.gustafson@colostate.edu (D.L.G.); 2 Department of Veterinary Clinical Sciences, College of Veterinary Medicine and Biomedical Sciences, The Ohio State University, Columbus, OH 43210, USA; quimby.19@osu.edu

**Keywords:** oral, ondansetron, pharmacokinetics, nausea, vomiting

## Abstract

**Simple Summary:**

Oral ondansetron is commonly prescribed to treat nausea and vomiting in dogs, but no studies have evaluated how well it is absorbed in clinical patients after oral administration. The objective of this study was to evaluate how well oral ondansetron is absorbed in a population of client-owned dogs with naturally occurring nausea. Twenty-four dogs were randomly assigned to receive one of the following doses of oral ondansetron, which are all within the currently recommended dose range: 0.5 mg/kg q8h, 0.5 mg/kg q12h, 1 mg/kg q8h, and 1 mg/kg q12h. Blood samples were collected for ondansetron measurement at various time points after administration of the first dose of ondansetron, and nausea scores were recorded. Ondansetron blood concentrations averaged over an 8 h time period were not significantly different between dose groups. The mean nausea scores at baseline were similar among all groups and decreased over time. Blood ondansetron concentrations were below the limit of detection in 44% (32/72) of all samples collected and were not detected at any timepoint in 25% (6/24) of the dogs. These results raise the concern that orally administered ondansetron at the current recommended dosages may not be absorbed into the bloodstream effectively.

**Abstract:**

The purpose of this study was to evaluate plasma ondansetron (OND) concentrations in a population of dogs with naturally occurring nausea after oral OND administration. Twenty-four dogs were randomly assigned to receive one of the following doses of oral OND: 0.5 mg/kg q8h, 0.5 mg/kg q12h, 1 mg/kg q8h, or 1 mg/kg q12h. Blood samples for plasma OND measurements were collected at baseline and 2, 4, and 8 h after administration of the first dose of OND. OND concentrations averaged over an 8 h time period were not significantly different between dose groups (0.5 mg/kg group: median 8.5 ng/mL [range 1–96.8 ng/mL], 1 mg/kg group: median 7.4 ng/mL [range 1–278.7 ng/mL]). The mean maximum concentrations in the 0.5 mg/kg and 1 mg/kg groups were 35.8 ± 49.0 ng/mL and 63.3 ± 121.1 ng/mL, respectively. OND concentrations were below the lower limit of quantification (LLOQ) in 50% (18/36) of samples in the 0.5 mg/kg groups and 39% (14/36) of samples in the 1 mg/kg groups. Six dogs (6/24, 25%) did not have OND detected at any time. The mean nausea scores at baseline were similar amongst all groups and decreased over time. The bioavailability of oral OND appears to be poor. Despite low plasma OND concentrations, nausea scores improved over time.

## 1. Introduction

Ondansetron (OND) is a 5-HT_3_ receptor antagonist that works through both central (5HT-3 receptors in the emetic center and chemoreceptor trigger zone) and peripheral (5HT-3 receptors on the vagal nerve terminals in the gastrointestinal tract) pathways to treat vomiting and nausea in veterinary patients [1,2,3]. However, OND is not currently approved for use in dogs for the treatment of vomiting and nausea. Currently, the only approved medication for the treatment of nausea and vomiting in dogs is maropitant citrate (Cerenia^®^, Pfizer Inc., East New York, NY, USA). Metoclopramide is another anti-emetic that is also not currently approved for use in dogs. Maropitant and metoclopramide have historically been used more commonly, and although both drugs can be effective anti-emetics [4,5,6,7,8,9,10,11,12,13,14,15,16,17,18], some studies have called into question how well they control nausea [7,11]. This is an important clinical consideration since the control of nausea is often the main therapeutic goal, as nausea can lead to not only vomiting but also ptyalism, lethargy, restlessness, and decreased appetite [1]. Therefore, a medication that is more effective at controlling nausea may be preferred over a medication that predominantly functions as an anti-emetic. OND has been shown in two studies to decrease or prevent vomiting, but it also appeared to be more effective than maropitant or metoclopramide at limiting nausea in the dog when given intravenously [7,11]. Both of these studies were performed using experimentally induced nausea models. The intravenous (IV) administration of OND has also recently been demonstrated to be effective at controlling nausea in dogs with vestibular syndrome [16,17].

Due to the previously published evidence that maropitant and metoclopramide may not be as effective as anti-nausea medications in dogs compared to OND, IV OND is commonly prescribed to dogs for control of vomiting and nausea. Additionally, there are very few anti-nausea medications that can be used for outpatient treatment in dogs. Oral maropitant can be cost-prohibitive for some clients, so oral OND is frequently prescribed. In a recent search at our facility, in 2023, our pharmacy recorded that 4 mg OND tablets were prescribed to 40 dogs and 8 mg OND tablets were prescribed to 170 dogs.

There is a paucity of literature on oral OND use in dogs experiencing nausea or vomiting. Recommended dosing of oral OND in dogs ranges from 0.2 to 1 mg/kg every 8 to 12 h [18]; however, this is not based on pharmacokinetic (PK) or pharmacodynamic data in dogs with naturally occurring vomiting and nausea. One study showed the severity and incidence of nausea were reduced in healthy dogs that received oral OND and were premedicated with glycopyrrolate, hydromorphone, and acepromazine; however, the incidence of vomiting was not reduced [15]. The bioavailability of oral OND is highly variable between species, including 4% in rats [19], 32% in cats [20], and 59% in humans [21]. In a population of healthy laboratory dogs, the oral bioavailability of OND was reported to be less than 10% [22]. Another veterinary study evaluated the PK of oral OND in 18 healthy beagles and reported high interindividual variability with an area under the curve (AUC) of 15.9 ± 14.7 ng·h/mL and a half-life of 1.3 h ± 0.7 h [23].

Plasma concentrations of OND after oral OND administration have not been evaluated in clinically ill dogs experiencing nausea or vomiting. The purpose of this study was to evaluate the plasma concentrations of OND after oral OND administration in a population of hospitalized dogs exhibiting clinical signs of nausea.

## 2. Materials and Methods

### 2.1. Sample Size Calculation

Using a variability of 60% in drug exposure, based on an oral OND PK study in cats [20], to show a significant difference of 1.3 to 2-fold in OND exposure based on a dose increase of 2-fold, 6 dogs per dosing group were required (24 dogs total) to achieve a power of >0.8 with *p* = 0.05. A sample size calculation was performed using Minitab software version 21.1.0 and one-way ANOVA analysis.

### 2.2. Animals

A prospective, randomized clinical trial was approved by the Colorado State University Clinical Review Board. Owners gave informed consent for their dogs to participate in the study. Dogs were enrolled from August 2021 through July 2022. Dogs presenting to the Colorado State University Veterinary Teaching Hospital were screened for enrollment. Eligible dogs were at least 6 months of age, at least 5 kg, and had a nausea score of at least 2, determined with a previously validated nausea scoring system [1,11,16,17]. Dogs were excluded if there was a documented or suspected small intestinal obstruction, if the dog had evidence of renal or hepatic disease, or if the dog received any anti-nausea medications in the previous 48 h. All dogs had a physical examination, a complete blood count, and a chemistry panel performed. Dogs had abdominal radiographs or an abdominal ultrasound performed at the discretion of the primary clinician.

### 2.3. Ondansetron Dosing and Blood Sample Collection

Dogs were randomized to one of four oral OND dosing groups: 0.5 mg/kg q8h, 0.5 mg/kg q12h, 1 mg/kg q8h, or 1 mg/kg q12h. The dose was based on actual body weight. Six dogs were enrolled in each group. All other medications were prescribed at the discretion of the primary clinician. OND was administered by members of the study team, and dogs were monitored to ensure the pill was swallowed. Blood samples were collected at baseline, 2, 4, and 8 h after administration of the first dose of OND in all dogs. Blood (1.3 mL) was collected in lithium heparin tubes. Samples were stored in a standardized-temperature refrigerator, and within 12 h of collection, all samples were centrifuged at 5500× *g* for 5 min. Plasma was then separated and immediately stored in polypropylene microcentrifuge tubes at −80 °C until later processing for full OND analysis.

### 2.4. Scoring Clinical Signs of Nausea

Nausea scores were assigned by a member of the study team using a previously validated nausea scoring table (Table 1) [1,11,16,17]. To improve the agreement of nausea scores between evaluators, the primary study investigators created a clinical scenario quiz involving 4 standardized cases where study team members assigned nausea scores. A quiz score of 100% was required before the scoring of clinical patients in the study could proceed. In patients dosed every 8 h, nausea scores were assigned at baseline, 4, and 8 h after the administration of the first dose. In patients dosed every 12 h, nausea scores were assigned at baseline, 4, and 12 h after the administration of the first dose. Nausea scores were assigned prior to blood sample collection. Whenever possible, baseline nausea scores and subsequent scoring were performed by the same team member.

### 2.5. Materials

OND (ondansetron hydrochloride, 4 mg and 8 mg tablets; Rising Health LLC; Saddle Brook, NJ, USA) was purchased through the pharmacy at Colorado State University Veterinary Teaching Hospital. Tablets were within the dated shelf-life, and they were stored as recommended.

### 2.6. Ondansetron Analysis

Plasma OND concentrations were measured using a liquid chromatography coupled to tandem mass spectrometry (LCMS) assay based on a previously published method [20], modified and previously published for the analysis of canine samples [24]. Samples were analyzed using an ABI 3200 QTrap triple quadrupole mass spectrometer (AB Sciex LLC, Framingham, MA, USA) with an Agilent 1200 LC system (Agilent Technologies, Santa Clara, CA, USA) and an HTC-Leap autosampler (CTC Analytics, Zwingen, Switzerland). Assay performance based on QC sample analysis at 3.9, 15.6, 62.5, and 250 ng/mL showed an accuracy and precision (CV%) of 90.4% ± 3.3%, with 16/20 (80%) QC samples showing an accuracy >85%. Batch acceptance was based on >75% of QC samples having an accuracy >85%, and the LLOQ for the analysis was 3.9 ng/mL based on the lowest concentration at least 2-fold above baseline and having an accuracy >80% [25]. OND concentrations below the LLOQ were set at 1 ng/mL for statistical analysis.

### 2.7. Plasma Ondansetron Concentrations and Statistical Analysis

The OND plasma concentration versus time data for each dog was subject to non-compartmental analysis using Phoenix WinNonLin v 8.3.4.295 (Certara, Princeton, NJ, USA). Statistical analysis was conducted using GraphPad Prism version 10.0.0 for MasOS, utilizing a 2-way ANOVA to assess time and dose, and comparisons between time points for the 0.5 and 1.0 mg/kg groups were performed using multiple Mann–Whitney tests. Values were considered significantly different if the *p*-value was <0.05.

## 3. Results

### 3.1. Animals

Twenty-four dogs that were hospitalized at the Colorado State University Veterinary Teaching Hospital were enrolled in the study. No dogs were removed from the study. None of the dogs vomited in the immediate timeframe after receiving the dose of OND. All dogs were inappetent at the time of evaluation and did not receive the medication with food. The mean age was 6.5 years (range 0.75–14.0 years), and the mean body weight was 22.6 kg (range 6.0–42.6 kg). The patient population included 15 (62.5%) males (13 castrated, 2 intact) and nine (37.5%) females (nine spayed and zero intact). The most commonly represented breeds included mixed breed dogs (9/24, 37.5%), Pit bull terriers or terrier mixes (3/24, 12.5%), Golden retrievers or retriever mixes (2/24, 8.3%), and German shepherds or shepherd mixes (2/24, 8.3%).

Primary diagnoses included gastritis, gastroenteritis, or colitis 13/24 (54%), acute hemorrhagic diarrhea syndrome 6/24 (25%), aspiration pneumonia 2/24 (8.3%), diabetic ketoacidosis 1/24 (4.2%), pancreatitis 1/24 (4.2%), and chemotherapy-induced nausea 1/24 (4.2%). The most commonly prescribed concurrent medications include IV fluids, gabapentin, probiotics, and trazodone.

### 3.2. Ondansetron Concentrations

**Figure 1:**
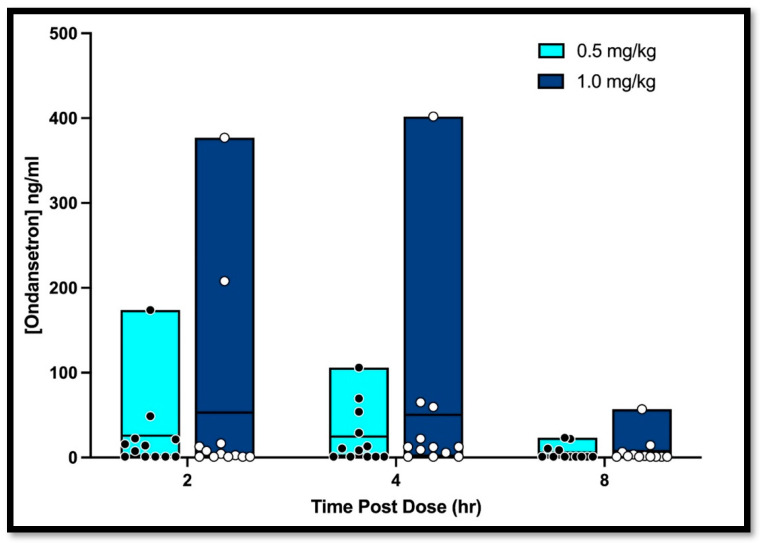
Median plasma ondansetron concentrations in dogs receiving oral ondansetron at 2, 4, and 8 h post-dose. Box and whisker plot representing minimum and maximum concentrations.

The median OND concentrations in the 0.5 mg/kg group at 2, 4, and 8 h were 10.84 ng/mL (range LLOQ—174 ng/mL), 9.63 ng/mL (LLOQ—106 ng/mL), and 1 ng/mL (LLOQ—23.4 ng/mL), respectively. The median OND concentrations in the 1 mg/kg group at 2, 4, and 8 h were 3.69 ng/mL (LLOQ—377 ng/mL), 12.3 ng/mL (LLOQ—402 ng/mL), and 1.32 ng/mL (LLOQ—57 ng/mL), respectively (Figure 1). There was no significant difference in the plasma concentration at 2, 4, or 8 h in the 0.5 vs. 1.0 mg/kg groups. The mean C_max_ in the 0.5 mg/kg and 1 mg/kg groups were 35.8 ± 49.0 ng/mL and 63.3 ± 121.1 ng/mL, respectively.

OND concentrations were below the LLOQ in 50% (18/36) of samples in the 0.5 mg/kg groups and 39% (14/36) of samples in the 1 mg/kg groups, totaling 44% (32/72) of samples. Six dogs (6/24, 25%) did not have OND detected at any timepoint. An equivalent number of dogs at both 0.5 and 1.0 mg/kg doses showed drug concentrations below the LLOQ across the sampling period.

### 3.3. Nausea Scores

**Figure 2:**
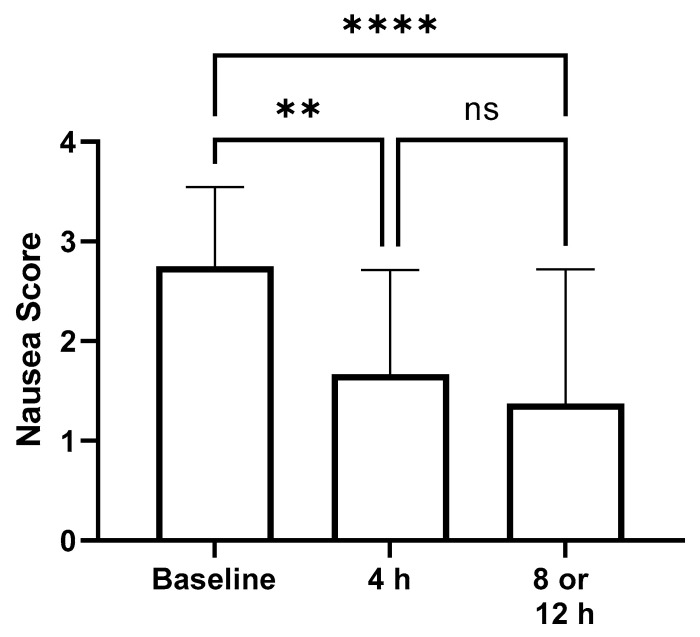
Mean nausea scores in dogs receiving oral ondansetron at baseline, 4, and 8, or 12 h. Plot represents mean with standard deviation. ** = statistical difference, *p* = 0.0003, **** = statistical difference, *p* < 0.001, ns = no statistical difference.

The mean nausea scores at baseline were similar among all groups. Mean baseline nausea scores at baseline in dogs receiving 0.5 mg/kg PO every 8 h and every 12 h were 3.0 (range 2–4) and 2.7 (range 2–3), respectively. Mean nausea scores at baseline in dogs receiving 1 mg/kg PO every 8 h and every 12 h were 3.3 (range 2–5) in both groups. When all 24 dogs were considered together, mean nausea scores at 4 h (1.4; 0–4) (*p* = 0.0003) and 8 or 12 h (0.92; 0–3) (*p* < 0.0001) were significantly decreased from baseline (3.1; 2–5) (Figure 2).

## 4. Discussion

This study evaluated the plasma concentrations of OND after oral OND administration in a population of dogs hospitalized with clinical signs of nausea, which had not been previously assessed. Dogs were randomly assigned to 0.5 mg/kg q8h, 0.5 mg/kg q12h, 1 mg/kg q8h, or 1 mg/kg q12h dosing groups, which are all within the currently recommended dose range [18]. Plasma OND concentrations were below the LLOQ in 44% (32/72) of all samples collected, and OND was not detected at any timepoint in 25% (6/24) of the dogs in this study.

The findings in the current study are consistent with the previously reported finding of low oral bioavailability of OND in laboratory dogs [22]. In the previous study, laboratory dogs receiving a single dose of 1 mg/kg of oral OND had less than 10% bioavailability of the drug, with a mean C_max_ of 8 ng/mL and a half-life of 30 min. Absorption of OND across the gastrointestinal tract was rapid, with a T_max_ of less than 40 min, and high first-pass metabolism was suspected to be the underlying cause of low systemic bioavailability. A more recent study found that a single oral 8 mg dose of OND (0.7–0.9 mg/kg dosing) administered to 18 healthy beagles resulted in a C_max_ of 11.5 ng/mL. This study found high interindividual variability with an area under the curve (AUC) of ±14.7 ng·h/mL and a half-life of 1.3 h ± 0.7 h [23]. Overall, highly variable OND PK was observed in the healthy dog population of this previous study, again attributed to low bioavailability secondary to significant first-pass metabolism. In the current study, dogs came from a heterogeneous population with different ages, weights, breeds, and diagnoses contributing to underlying clinical signs of nausea. In the current study, intersubject variability and suspected low oral bioavailability may explain why OND concentrations were below the LLOQ in 50% of samples in the 0.5 mg/kg groups, 39% in the 1 mg/kg groups, and non-detectable in 25% of dogs at any timepoint. To better evaluate the oral bioavailability of OND, a crossover study evaluating the PK of both IV and oral OND in a single population of dogs is warranted.

The OND concentrations after oral OND administration in dogs demonstrated in the current study can also be compared to previous PK studies in laboratory rats, healthy cats, and humans. In laboratory rats, oral OND administered at 1 mg/kg resulted in a C_max_ of 15 ng/mL and was shown to have poor systemic bioavailability (<10%) [22]. A more recent study found that oral OND administered at 8 mg/kg in rats resulted in a C_max_ of 0.31 µg/mL and an oral bioavailability of 4% [19]. The findings of poor systemic bioavailability in rats are similar to those in dogs and are also suspected to be secondary to high first-pass metabolism and high systemic clearance of the drug [19,22]. In healthy cats, oral OND administered at 0.43 mg/kg resulted in a C_max_ of 92.7 ng/mL and was reported to be more bioavailable (32%) as compared to the dog or rat [20]. However, oral bioavailability was significantly less than the bioavailability of subcutaneously administered OND (72%) [20]. In contrast, in humans, bioavailability has been demonstrated to be 59%, which likely accounts for the popularity of its use to treat vomiting and nausea in human patients [21].

In this study, the mean C_max_ of oral OND in the 0.5 mg/kg and 1 mg/kg groups was 35.8 ng/mL and 63.3 ng/mL, respectively. A recent study looking at the PK of IV OND in hospitalized dogs exhibiting clinical signs of nausea found that the mean C_max_ of OND in the 0.5 mg/kg and 1 mg/kg groups was 214 ng/mL and 541 ng/mL, respectively [24]. Notably, in the previous study, OND reached measurable concentrations in all dogs, and these concentrations were within the estimated therapeutic range for the dosage regimens used in the study [24]. The lack of detection of plasma OND concentrations in samples collected and lower plasma concentrations in comparison to the previously performed IV study raises concern for using OND via the oral route in this population of dogs. Other potential causes of low plasma OND concentrations, including improper dosing, administration with food, and vomiting immediately after administration, are unlikely, as all patients were dosed by experienced personnel and monitored to ensure the pill was swallowed, none of the dogs were willing to eat at the time of medication administration, and none of the dogs vomited in the immediate timeframe after dosing.

Assessing nausea in veterinary patients can be very difficult and is generally considered a subjective assessment. Arginine vasopressin (AVP) has been assessed as a biomarker for nausea in dogs, with mixed results. In a previous study using healthy beagles, AVP concentrations increased after the administration of cisplatin, a known emetogen [11]. In the same study, the administration of IV OND prevented the increase in AVP [11]. In a more recent study, AVP levels decreased as compared to baseline after the administration of IV OND in dogs with vestibular syndrome [17]. However, in a study of dogs hospitalized with clinical signs of nausea and receiving IV OND, AVP concentrations were highly variable and did not correlate with nausea scores [24]. In addition to the mixed results, AVP is not a readily accessible test, so its clinical value would be limited. In an effort to reduce the subjectivity associated with assessing nausea, nausea scoring systems have been created and validated [1,11,16,17]. They too have limitations, as there is still some subjective assessment required and interobserver variability has not been assessed. We attempted to reduce the risk of interobserver variability in this study by limiting the number of evaluators and providing training to all evaluators.

In the current study, nausea scores significantly decreased from baseline, regardless of oral OND dosage. It is important to note that other therapies were being concurrently administered to each dog while hospitalized. Examples of concurrent therapies include IV fluids to correct clinically apparent dehydration, antimicrobials to address suspected underlying bacterial infections, and pain medications. Therefore, correcting dehydration and instituting needed therapies may have been the cause of the decrease in clinical signs of nausea rather than the OND alone, or it was a combination of all supportive care strategies. Although the population of dogs studied was heterogeneous and was receiving multiple therapies, which could be considered a limitation of the study, this cohort of dogs represents typical clinical practice.

Another explanation for decreasing nausea scores despite low or undetectable plasma OND concentrations is that the anti-emetic effects of OND may not be best reflected by plasma concentrations but rather pharmacodynamic parameters. It is possible that oral OND is exerting anti-nausea and anti-emetic effects locally within the gastrointestinal tract [2]. A previous study showed that IV OND had anti-emetic effects when evaluating a peripheral emetogen, syrup of ipecac, but was not very effective when evaluating a central emetogen, apomorphine [5]. There are a multitude of serotonin receptors in the gastrointestinal tract, and serotonin receptors and serotonin release can be altered in humans with various intestinal diseases [26,27,28,29]. Serotonin receptor antagonism via oral OND, which is rapidly absorbed by the gastrointestinal tract, could be why patients improved in nausea scores since many of the dogs were suffering from suspected direct gastrointestinal inflammation such as gastroenteritis and acute hemorrhagic diarrhea syndrome. Future studies investigating the PK and pharmacodynamics of oral OND in dogs discharged for outpatient care are warranted to help better address this clinical query.

The primary limitation of this study was the limited number of samples collected, which was further limited by the high number of samples (32/72, 44%) where OND concentrations were below the LLOQ. The limited samples available for analysis may have resulted in a type II error. In addition, this study did not include a control group because the primary goal was to evaluate the plasma concentrations of OND. Another potential limitation to this study is that the population of dogs studied was heterogeneous; however, this cohort of dogs represents that of typical clinical practice and a population for which OND would be prescribed.

In conclusion, OND is a frequently prescribed anti-emetic and anti-nausea drug in veterinary medicine. IV administration of OND has demonstrated efficacy as an anti-emetic in several populations of nauseous dogs, and oral administration has been shown to reduce the incidence of severe nausea but not vomiting in a population of healthy dogs [15]. The PK of oral OND has been investigated previously in healthy dogs, but this is the first study investigating the plasma concentrations in a population of hospitalized dogs exhibiting clinical signs of nausea. In this study, OND concentrations were below the LLOQ in 44% (32/72) of all samples collected. In addition, OND was not detected at any timepoint in 25% (6/24) of the dogs in this study. The bioavailability of oral OND appears to be poor. Despite low plasma OND concentrations, however, the mean nausea scores decreased over time. Nausea scores may have improved due to concurrent therapies, including IV fluids and pain medications. Alternatively, this may suggest that the anti-emetic and anti-nausea effects of OND may not be correlated with plasma OND concentrations. Further evaluation of the pharmacodynamics of oral OND in clinically ill dogs should be considered.

## Figures and Tables

**Table 1:** Nausea Scoring System in Dogs.

Behavior	Description	Score
Salivation	Drooling, increase in swallowing frequency, gulping, chewing movements	1
Gastrointestinal Disturbances	Pica, belching, productive or nonproductive vomiting	1
Lip Smacking	Lip licking, licking the nose, nictitation (winking)	1
Excitement Behaviors	Apprehension, restlessness, rapid breathing	1
Vocalization	Murmuring, groaning, whining	1
Withdrawal Behaviors	Standing with head drooping, closing eyes, yawning, drowsiness, lethargy, and excessive sleeping	1
Appetite	Decreased appetite, avoidance of food bowl	1

Table references from Kenward, Hannah et al. “Nausea: current knowledge of mechanisms, measurement and clinical impact.” *The Veterinary Journal* 203.1 (2015): 36–43 and Kenward, Elliot, et al. “Anti-nausea effects and pharmacokinetics of ondansetron, maropitant and metoclopramide in a low-dose cisplatin model of nausea and vomiting in the dog: a blinded crossover study.” *BMC veterinary research* 13.1 (2017): 1–12.

## Data Availability

The raw data supporting the conclusions of this article will be made available by the authors on request.
